# Racial differences in endometrial cancer molecular portraits in The Cancer Genome Atlas

**DOI:** 10.18632/oncotarget.24907

**Published:** 2018-03-30

**Authors:** David S. Guttery, Kevin Blighe, Konstantinos Polymeros, R. Paul Symonds, Salvador Macip, Esther L. Moss

**Affiliations:** ^1^ Leicester Cancer Research Centre, University of Leicester, Robert Kilpatrick Building, Leicester Royal Infirmary, Leicester, UK; ^2^ Department of Gynaecological Oncology, University Hospitals of Leicester NHS Trust, Leicester General Hospital, Leicester, UK; ^3^ Mechanisms of Cancer and Ageing Lab, Department of Molecular and Cell Biology, University of Leicester, Leicester, UK

**Keywords:** endometrial cancer, ethnicity, TCGA, somatic copy number aberrations

## Abstract

Endometrial cancer (EC) is now the most prevalent gynaecological malignancy in the Western world. Black or African American women (BoAA) have double the mortality of Caucasian women, and their tumours tend to be of higher grade. Despite these disparities, little is known regarding the mutational landscape of EC between races. Hence, we wished to investigate the molecular features of ECs within The Cancer Genome Atlas (TCGA) dataset by racial groupings. In total 374 Caucasian, 109 BoAA and 20 Asian patients were included in the analysis. Asian women were diagnosed at younger age, 54.2 years versus 64.5 years for Caucasian and 64.9 years for BoAA women (OR 3.432; p=0.011); BoAA women were more likely to have serous type tumors (OR 2.061; p=0.008). No difference in overall survival was evident. The most frequently mutated gene in Caucasian and Asian tumours was *PTEN* (63% and 85%), unlike BoAA cases where it was *TP53* (49%). Mutation and somatic copy number alteration (SCNA) analysis revealed an enrichment of *TP53* mutations in BoAAs; whereas *POLE* and *RPL22* mutations were more frequent in Caucasians. Major recurrent SCNA racial differences were observed at chromosomes 3p, 8, 10, and 16, which clustered BoAA tumors into 4 distinct groups and Caucasian tumors into 5 groups. There was a significantly higher frequency of somatic mutations in DNA mismatch repair genes in Asian tumours, in particular *PMS2* (p=0.0036). In conclusion, inherent racial disparities appear to be present in the molecular profile of EC, which could have potential implications on clinical management.

## INTRODUCTION

Endometrial cancer (EC) is the most common gynaecologic malignancy in the Western world. In the USA in particular, it is the fourth most common cancer in females, with rising incidence and reduced survival [1]. There is strong evidence to suggest that race has an influence in the prognosis of the disease. Black women have a 19-23% lower risk of developing the disease over their lifetime; however, EC incidence in black women has been increasing and their rates even surpass those of Caucasians when correcting for hysterectomy [2, 3]. Furthermore, they tend to have worse outcome and almost double the mortality rate of Caucasian women [4], along with much higher incidence of Type 2 tumors [5], conferring a significantly worse prognosis. Many factors have been proposed as contributing to the poor outcome observed in Black or African American (BoAA) women, including socioeconomic, biologic, access to healthcare and treatment [6]. Among biologic factors, higher prevalence of *PTEN* mutations in Caucasian women has been reported [7] and these are considered to offer better prognosis [8]. Microsatellite instability (MSI) is three times more common in the tumors of Caucasians compared to BoAA women [9]; whereas BoAA women have higher incidence of *TP53* mutations [10], thought to be related to higher incidence of Type 2 tumors in these patients. Racial differences have also been seen with *PIK3CA* and *KRAS* mutations being seen more frequently in BoAA women as compared to Caucasian women in low-grade endometrioid EC [11]. Unlike the breadth of knowledge regarding the comparison between BoAA and Caucasian women, much less is known about other ethnic minority groups, for example, Asian. The incidence of EC is almost 30% less in this group compared to Caucasians [1] and women tend to present younger at diagnosis but are reported to have a slightly better outcome [12].

Despite these disparities, little is known regarding the mutational landscape of EC between races. To address this, we investigated the molecular features of EC, including somatic mutations, gene expression, and copy-number aberrations (CNAs) and contrasted them between tumours from BoAA, Caucasian and Asian women to examine differences in EC disease/molecular profile and overall survival.

## RESULTS

### Population-based analysis

The TCGA dataset comprised of 374 Caucasian, 109 BoAA, and 20 Asian patients. Using a multinomial logistic regression model and Caucasians as the reference model: Asian patients were diagnosed at a significantly younger age (OR 3.43; 95% CI, 1.34-8.6; p=0.011). BoAA women were more likely to be diagnosed with serous-type tumors (OR 2.06; 95% CI 1.25-3.36; p=0.008) and have higher BMI (OR 2.94; 95% CI, 1.42-6.89; p=0.0008) (Table 1). There was no significant difference in overall or progression-free survival between races using a Cox-proportional hazards model.

**Table 1:** Description of endometrial cancer cohort

Characteristic	Sub-category	Caucasian (n=374) N (%)	Asian (n=20) N (%)	p	OR (95% CI)	BoAA (n=109) N (%)	p	OR (95% CI)
Age at diagnosis	≤55	72 (19.3)	9 (45)	**0.011**	3.432 (1.338-8.6)	16 (14.7)	0.27	0.722 (0.389-1.272)
	>55	302 (80.7)	11 (55)	-	-	93 (85.3)	-	-
BMI	Obese	211 (56.4)	7 (35)	0.19	0.351 (0.1164-NA)	67 (61.5)	**0.0008**	2.937 (1.420-6.887)
	Overweight	73 (19.5)	6 (30)	-	0.869 (0.268-NA)	22 (20.2)	-	2.788 (1.207-7.042)
	Underweight	3 (0.8)	0 (0)	-	NA	0 (0)	-	NA
	Unknown	13 (3.5)	0 (0)	-	NA	12 (11)	-	8.539 (2.988-25.966)
	Normal	74 (19.8)	7 (35)	-	-	8 (7.3)	-	-
Clinical stage	I	245 (65.5)	14 (70)	-	-	57 (52.3)	-	-
	II	33 (8.8)	3 (10)	-	1.061 (0.162-4.025)	12 (11.)	-	1.563 (0.735-3.144)
	III	79 (21.1)	2 (15)	-	0.665 (0.15-2.1)	32 (29.4)	-	1.741 (1.047-2.864)
	IV	17 (4.6)	1 (5)	0.93	1.029 (0.055-5.615)	8 (7.3)	0.094	2.023 (0.792-4.79)
Histologic grade	G1	75 (20.1)	6 (30)	-	-	14 (12.8)	-	-
	G2	88 (23.5)	4 (20)	-	0.568 (0.141-2.063)	26 (23.9)	-	1.583 (0.781-3.32)
	G3	211 (56.4)	10 (50)	0.59	0.592 (0.213-1.793)	69 (63.3)	0.2	1.803 (0.995-3.41)
Histologic type	Endometrioid	293 (78.3)	17 (85)	-	-	69 (63.3)	-	-
	Serous	68 (18.2)	3 (15)	0.46	0.76 (0.174-2.344)	33 (30.3)	**0.008**	2.061 (1.252-3.356)
	Mixed	13 (3.5)	0 (0)	-	NA	7 (6.4)	-	2.287 (0.832-5.803)

Characteristics showing evidence of racial disparity included age at diagnosis, BMI, and histologic type (p<0.05), but no statistically significant differences for histologic grade. Clinical stage exhibited a trend toward racial disparity. All p values and odds ratios (ORs) are derived from χ^2^ ANOVA on a multinomial logistic regression model with race as outcome, with levels ordered as Caucasian, BoAA, and Asian. Reference categories for reference models: race, Caucasian; age at diagnosis, >55; BMI, normal; clinical stage, stage I; histologic grade, G1; histologic type, endometrioid.

### RNA and microRNA expression

Tumor histologic type/grade, clinical stage, and BMI were statistically significant drivers of variation in the RNA-seq expression data (Spearman's Rho to PC1 eigenvalues; *P*<0.001). In miRNAs, the same plus age at diagnosis were statistically significant at the same level of significance but to PC2. 4933 transcripts (8.52%) were identified as differentially expressed between tumour and normal samples. Of the 4933 transcripts differentially expressed in tumours compared to normal tissue, pairwise comparisons between each race then revealed 44 transcripts (0.076%) differentially expressed between BoAAs and Caucasians, 131 (0.23%) between BoAAs and Asians, and 78 (0.14%) between Asians and Caucasians (Supplementary Tables 1-3). The top differentially expressed genes for each group were *UTF1* in BoAA tumors compared to Caucasian; *SLC14A2* in BoAA compared to Asian tumors and *GSTA1* in Asian tumors compared to Caucasian.

Two hundred and eighty miRNAs (17.24%) were identified as differentially expressed between tumour and normal samples. Pairwise comparisons of miRNAs differential expressed between tumors and normal samples were then compared in each race, filtering on an FDR Q< 0.05 and log fold change >2. Expression of a single miRNA (miR1269b) was significantly increased in BoAA tumors compared to both Caucasian and Asian tumors. Three miRNAs (miR1269a, miR891a and miR892a) were significantly decreased in Asian tumors compared to Caucasian tumors.

### Somatic mutations and recurrent somatic copy number alterations (SCNA)

**Figure 1:**
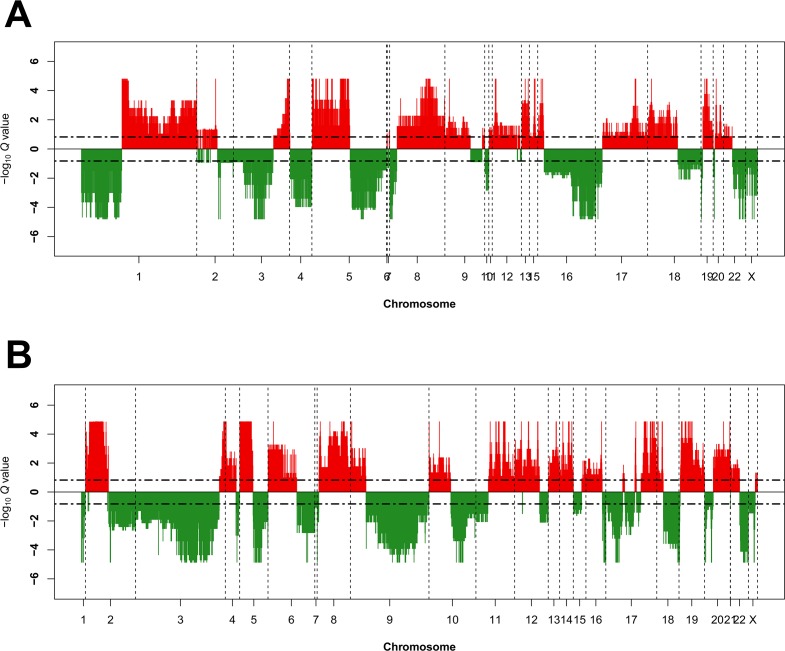
Recurrent genome-wide SNCAs in each race Genome-wide amplifications and deletions in BoAAs **(A)** and Caucasians **(B)**. Recurrent SCNA in the GISTIC 2.0 SCNA data was calculated using GAIA [42] with known common CNV filtered out. Recurrent CNV were defined by FDR Q<0.15 using ten iterations. Genomic SCNA plots were generated using a custom R script, with cut-off defined also at FDR Q<0.15 for the purposes of visualisation. Large genomic differences in recurrent SCNA profiles were observed between each race.

Excluding variants identified in an unpaired panel of normals, 833,034 mutations were identified including 604,192 (1,669/tumor) in Caucasian tumors, 136,009 (1,283/tumor) in BoAAs, and 92,833 (4,642/tumor) in Asian samples. The most frequently mutated gene in tumors from Caucasian and Asian women was *PTEN* (63% and 85%, respectively); however, the specific mutations they contained differed in frequency: *PTEN* p.Arg130Gly was more common in tumors from Caucasian women and *PTEN* p.Arg130Gln in Asian cases (Supplementary Table 4). The most frequently mutated gene in BoAAs was *TP53* (49%) whereas *KRAS* p.Gly12Asp was the most frequent individual mutation. Of note, observing mutation frequencies across the 12 top mutated genes from the original TCGA study (i.e., *PTEN*, *PIK3CA*, *ARID1A*, *TP53*, *CTNNB1*, *CTCF*, *KRAS*, *PIK3R1*, *FBXW7*, *PPP2R1A*, *ARID5B*, and *RPL22*), we observed statistically significant differences across ethnicities (χ^2^ P=0.002) (Table 2). For SCNA analysis, clear major chromosomal abnormalities existed between Caucasian and BoAA tumors, including recurrent deletions at chromosome 3p in Caucasians and amplification of chr1q and deletions in chr16 in BoAAs (Figure 1).

**Table 2:** Recurrently mutated genes differing by ethnicity

Caucasian		Asian		BoAA	
Gene	n (%)	Gene	n (%)	Gene	n (%)
*PTEN*	229 (63.26)	*PTEN*	17 (85)	*TP53*	52 (49.06)
*PIK3CA*	181 (50)	*PIK3CA*	13 (65)	*PTEN*	41 (38.68)
*ARID1A*	159 (43.92)	*ARID1A*	9 (45)	*PIK3CA*	41 (38.68)
*TP53*	115 (31.77)	*PIK3R1*	6 (30)	*ARID1A*	30 (28.3)
*CTNNB1*	98 (27.07)	*ARID5B*	6 (30)	*FBXW7*	24 (22.64)
*CTCF*	86 (23.76)	*CTNNB1*	6 (30)	*CTCF*	20 (18.87)
*KRAS*	76 (21)	*TP53*	5 (25)	*PIK3R1*	18 (16.98)
*PIK3R1*	71 (19.61)	*KRAS*	5 (25)	*CTNNB1*	17 (16.04)
*FBXW7*	64 (17.68)	*CTCF*	5 (25)	*KRAS*	15 (14.15)
*PPP2R1A*	62 (17.13)	*FBXW7*	3 (15)	*PPP2R1A*	13 (12.26)
*ARID5B*	50 (13.81)	*RPL22*	3 (15)	*ARID5B*	12 (11.32)
*RPL22*	41 (11.33)	*PPP2R1A*	2 (10)	*RPL22*	8 (7.55)

Next, the relationship of race with the 4 TCGA groups (*POLE* ultramutated, microsatellite instability (MSI) hypermutated, copy-number low, and copy-number high) [13] was investigated. Note that Asian women were removed from this analysis due to small numbers of cases.

**Figure 2:**
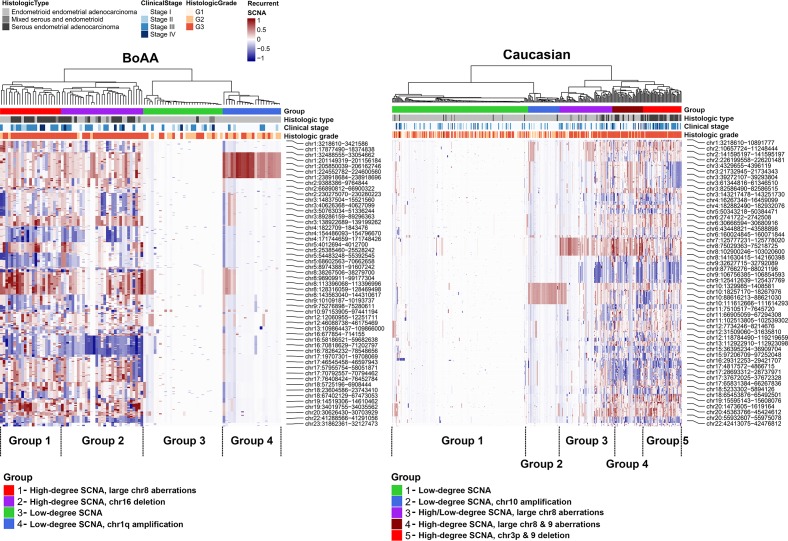
Heatmap of significant SCNA groups in BoAA and Caucasians Clustering was performed on the copy number segment mean for each recurrent SCNA region passing FDR Q<0.15. Dendrograms were generated using Euclidean distance and Ward's linkage. To identify groups of SCNA profiles in each race, we cut the dendrogram tree at different heights in order to isolate groups that fit the patterns of SCNA in the heatmap. For heatmap shading, we used a 100-element colour palette of darkblue-to-white-to-darkred and set breaks at −1 and+1.

Through hierarchical clustering, 4 distinct groups were identified within the BoAA tumors (2 high-degree SCNA and 2 low-degree SCNA) and 5 in the Caucasian cases (2 high-degree SCNA, 1 high/low degree SCNA and 2 low-degree SCNA) (Figure 2). In both races, groupings segregated with histologic type/grade and clinical stage, with serous (BoAA, P=0.00435; Caucasian, P<0.0001), stage III (Caucasian, P=0.00469), and grade 3 (BoAA, P=0.000467; Caucasian, P<0.0001) tumors exhibiting a higher degree of SCNA. One group, seen in both Caucasian (Group 1) and BoAA (Group 3) tumors, was almost entirely absent of statistically significant recurrent SCNA, mainly segregating with endometrioid, stage I, and low-grade tumors.

**Figure 3:**
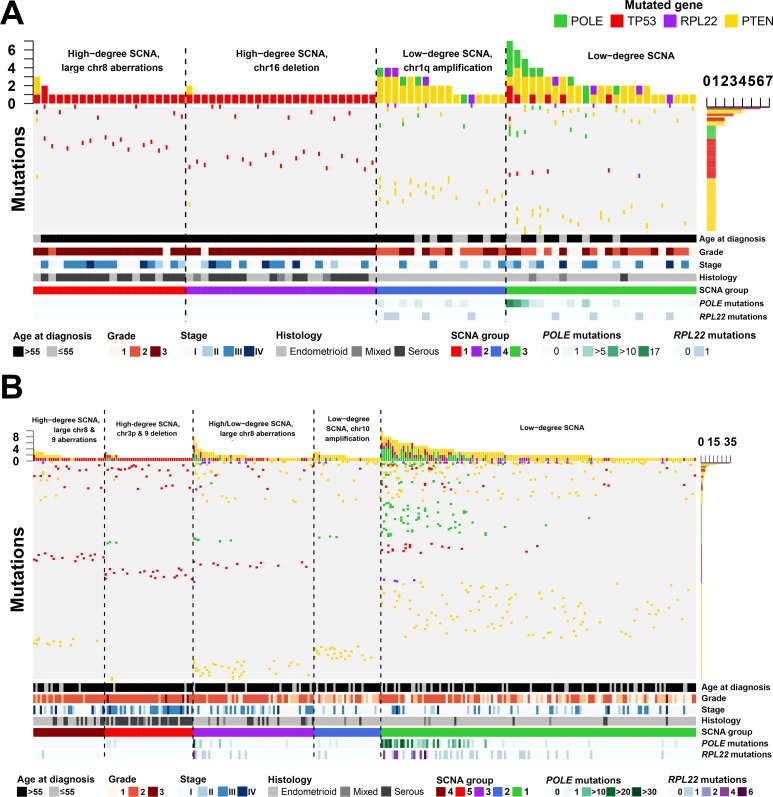
Mutational profiles across endometrial cancers in BoAA and Caucasian patients Mutation frequencies (vertical axis) are plotted for each tumor (horizontal axis) for BoAAs **(A)** and Caucasians **(B)**. Details are given regarding patients demographics. Only patients with mutations are illustrated. See Supplementary Table 5 for full details of cohort numbers and groupings.

Comparing the average overall mutations/sample, *POLE* and *RPL22* were more frequently mutated in Caucasian compared to BoAA cases (0.285 versus 0.179, and 0.114 versus 0.085 mutations/sample respectively). The relationship between SCNA groupings and mutation data identified a group in BoAA cases (Group 1), which was dominated by serous tumors (P<0.0001), exhibited mainly *TP53* mutations and a had complete absence of *POLE* or *RPL22* mutations (Figure 3A); whereas the other high-degree SCNA group (Group 2) was dominated by different *TP53* mutations and also serous tumours (P=0.000374). Both low-degree SCNA groups in BoAAs were almost entirely endometrioid tumors dominated by *PTEN* mutations. The profiles for the Caucasian cases were quite different, with all SCNA groups harbouring *TP53* mutations and Group 5 (High degree SCNA, chr3 and 9 deletion) dominated by serous tumours. The Caucasian tumors contained a sub-group of cases with a high *POLE* mutation rate (Group 1), which corresponded to the *RPL22*-mutated sub-group. All the subgroups contained different *PTEN* mutations, with the exception of Group 5, where *PTEN* mutations were mostly absent (Figure 3B). Across all SCNA groups in both BoAA and Caucasian tumors, *TP53*, *POLE* and *RPL22* mutation counts, were significantly different by ANOVA (P<0.05). For most common mutations per group, per race - see Supplementary Table 5.

**Figure 4:**
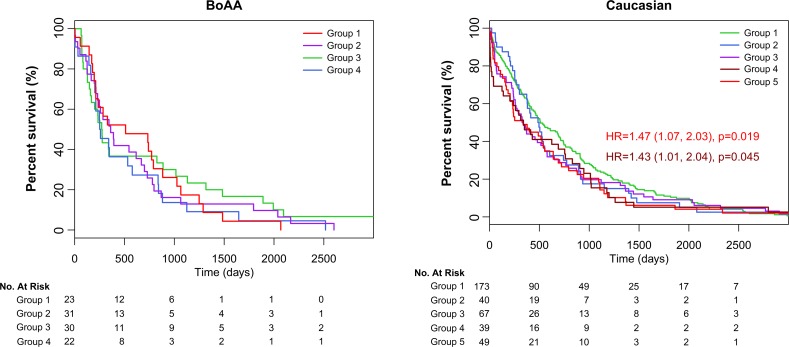
Survival analysis of each group Kaplain-Meier curves by ethnicity showing 2 groups with significantly poorer outcome in Caucasians.

No significant differences in overall survival was observed between any of the BoAA groups. However, in the Caucasian cases, two groups exhibited statistically significant worse overall survival, both of which harboured high-degree SCNAs: Group 4 (dominated by *PPP2R1A* mutations (18% of patients in Group 4). HR=1.43; 95% CI, 1.01-2.04; *P*=0.045) and Group 5 (dominated by *TP53, PPP2R1A* and *GOLGA6L6* mutations. HR=1.43; 95% CI, 1.01-2.04; *P*=0.045) (Figure 4).

### DNA mismatch repair genes

Lynch syndrome (LS) is one of the most common inherited causes of endometrial cancers [14]. However, in many cases immunohistochemical and/or MSI testing suggests LS but further investigation shows no evidence of germline mutations, with somatic mutations being attributed to up to 70% of mismatch repair (MMR)-deficient colon and endometrial cancers [15]. Analysis of somatic mutations in MMR-associated genes identified an enrichment of somatic mutations in *MLH1, MSH2, MSH6 and PMS2* across the 3 racial groups with a higher frequency of mutations in the Asian cases, predominantly missense mutations but also nonsense and silent (Supplementary Figure 1). In particular, *PMS2* mutations were found to be significantly enriched (Fisher's exact test; p=0.0036). Unfortunately, data was not available on the presence of germline MMR mutations and therefore we are not able to comment on the incidence of LS in this cohort.

## DISCUSSION

Analysis of the genomic profile of endometrial carcinomas within the TCGA database has led to ground breaking advances in our understanding [13], in particular with the identification of four distinct overarching subtypes. The analysis we have conducted has furthered this understanding by identifying significant differences in genomic profiles between different races, which have potential implications not only for the classification of tumors but also with clinical management by helping to select optimum treatment regimens for patients, and advising on prognosis/screening.

Our results indicate that the four TCGA groups should be viewed as a top-level classification and that performing analysis by race gives additional subgroups within the Caucasian and BoAA populations. It is well recognised that BoAA EC patients have significantly poorer survival compared to Caucasians, which is attributed to a greater proportion of advanced stage, serous-like cancers in the BoAA population [16, 17]. Serous-like EC is associated with *TP53* mutations and falls within the TCGA copy number high group. In our analysis we have shown that *TP53* mutations were seen more frequently in the BoAA population and although this is not surprising, given the increased prevalence of serous-like cancers in this population, we have also identified two distinct high-copy number subgroups in the BoAA population. The BoAA Group 1 contains high-degree SCNA, large aberrations on chromosome 8 and 2 *TP53* mutations (p.Arg273His and p.Arg248Gln), which are known to be common in Type 2 tumors (and thereby confer a poorer prognosis) [18]. The other high SCNA group (Group 2) contains only the *TP53* p.Arg273His mutation, but not the additional p.Arg248Gln. It is known that different *TP53* mutations confer different functions to the protein in cancer [19]. The *TP53* p.Arg273His mutant is known to bind the MRE11 nuclease, resulting in increased genomic instability [20]. However, the *TP53* p.Arg248Gln mutant has been associated with metastasis in mouse models [21] and shorter patient survival in an analysis of TCGA data for breast invasive carcinoma, colorectal cancer, glioblastoma, lung squamous cell carcinoma and ovarian serous cystadenocarcinoma [22]. The same study also showed patients harbouring *TP53* p.Arg273His mutations have similar survival curves to patients with nonsense mutations. Therefore, it is reasonable to hypothesise that in these 2 groups, the *TP53* p.Arg273His mutant may be driving their genomic instability, but it is the *TP53* p.Arg248Gln mutation that determines their prognosis.

The finding of a subgroup of BoAA patients harbouring amplification of chr1q is interesting since a recent study has shown amplification at chr1q21.3 to be a biomarker for breast cancer relapse [23]. This region encodes a number of S100A-family proteins, which have been shown to drive tumorsphere growth via a reciprocal feedback loop that can be disrupted using the JAK inhibitor, Pacritinib [23]. Furthermore, they have also been shown to activate the epithelial-to-mesenchymal phenotype and promote cell migration and invasion [24]. This therefore may present a personalised approach for treatment of patients harbouring this amplification.

In Caucasian SCNA groups with significantly poorer survival (Groups 4 and 5), both groups contained a number of *PPP2R1A* mutations including p.Pro179Arg, p.Ser256Phe and p.Trp257Leu, all 3 of which have been implicated in promoting malignant cell growth [25]. Furthermore, the p.Trp257Gly mutation has also been shown to increase cancer cell migration through the SRC-JNK-c-Jun pathway [26]. *PPP2R1A* mutations are known to be enriched in serous EC tumors, and therefore it is not surprising to see the 2 groups enriched for these mutations are predominated by serous ECs. Group 5 also contained a number of *GOLGA6L6* mutations. To our knowledge, no literature exists detailing any of the mutations highlighted in this study and may be a novel avenue for investigation.

Differential expression was observed in key EC-associated genes between the Caucasian and BoAA cases; however, our analysis has identified several genes that have not been previously recognised to play a role in EC carcinogenesis. *UTF1* had the greatest differential RNA expression between the BoAA and Caucasian populations. Increased expression of *U*TF1 (resulting from hypermethylation of its promoter) has been suggested as a biomarker of cervical cancer diagnosis, with inhibition of DNA methyltransferase by 5-aza-2′-deoxycytidine (also known as Decitabine) reducing UTF1 gene methylation and expression in two cervical cancer cell lines [27]. Furthermore, SLC14A2, which was significantly higher in both BoAA and Caucasian tumours compared to Asian cases has been shown to be down-regulated in chemotherapy-resistant ovarian cancer cell lines [28], suggesting potential race-specific targets for treatment. The estrogen-target gene TFF1 was upregulated in BoAA tumors. TFF1 is known to play a role in breast cancer development [29] and bone metastasis [30], has also been suggested as a poor prognostic indicator associated with lymph node metastasis in pancreatic cancer [31]. A previous study has shown that a combination of the progestin medroxyprogesterone acetate (MPA) and the Ras inhibitor S-farnesylthiosalicylic acid (FTS, also known as Salirasib) inhibited tumor growth and enhanced type 2 EC cell death by reducing expression of ER-target genes, including TFF1 [32]. Since a large proportion of BoAA tumors are type 2 ECs, this presents a promising strategy for therapy in these patients.

BoAA tumours had higher levels of miR1269b, which has been shown to be increased in hepatocellular carcinoma (HCC) and promotes HCC cell growth by down-regulating FOXA1 [33]. Furthermore, miR1269b has been shown to target and enhance expression of the cell cycle regulator CDC40, known to mediate proliferation and migration of HCC cells [34]. This finding presents another novel target for therapeutic intervention in the BoAA population.

The high frequency of somatic mutations in MMR genes in the Asian compared to the Caucasian/BoAA groups is potentially of clinical importance, and could possibly explain the much higher number of mutations in the Asian cohort and the younger age at diagnosis. Somatic mutations of MMR genes can arise in cases without a germline mutation and although hypermethylation of MLH1, EPCAM germline mutations and MSH2 inversions have been identified as likely causes in the majority of cases they do no explain all the cases [35, 36]. In a recent study, deficiency in mismatch repair pathways has been shown to predict response of solid tumours (including endometrial cancers) to PD-1 blockade [37]. Furthermore, epithelial ovarian cancers with MMR-deficiencies were more sensitive to the PARP inhibitor Olaparib [38]. This offers a promising treatment strategy for patients enriched for MMR-deficient ECs and indeed, treatment for MSI-high or MMR-deficient tumours has recently been approved by the FDA.

### Limitations

The primary limitation of this study is the unequal number of cases from the different racial groups within the cohort. In some analyses this has resulted in insufficient statistical power between the three groups, for example to confirm a significant difference between MMR gene mutations between the Asian and Caucasian/BoAA populations. Also, the terms ‘Caucasian’, ‘BoAA’ and ‘Asian’ encompasses people from huge geographical areas and since there is a lack of sub-regional information on the cases included in the dataset it is not possible to determine whether certain populations may be over represented in each group. Further work is needed with larger patient numbers to determine whether the genetic profile seen particularly in the Asian group is representative of this population as a whole or whether defined subpopulations are at greater risk. Furthermore, admixture is likely present and unable to be accounted for in this dataset, but may introduce variability. Thirdly, the confounding factor of medical insurance and socioeconomic status on cancer outcomes has been reported previously and potentially could have an impact on patient survival. However, treatments received, age, type of surgery, etc. could all affect overall survival, and are unadjusted for in this study. Despite this, our results support findings from other studies of a racial disparity in EC survival.

## CONCLUSIONS

In summary, we have identified clear differences in the molecular portraits in EC from Caucasian, BoAA and Asian patients. The results have implications for patient management by enabling tumors to be classified into subgroups, in addition to the four TCGA groups, that carry significant prognostic information.

## MATERIALS AND METHODS

### Study cohort

Open access data on Uterine Corpus Endometrial Carcinoma was accessed (01/07/2017) from The Cancer Genome Atlas (TCGA) https://portal.gdc.cancer.gov/ [13]. Race was categorised according to the TCGA groupings of Caucasian, Black or African American (BoAA) and Asian. Other racial groups were excluded due to the small patient numbers. Somatic copy number alteration (SCNA) data minus germline SCNA was produced using GISTIC 2.0 from the Broad Institute of Harvard and MIT's FireBrowse (http://firebrowse.org/). Raw data was produced as described previously [13].

### RNA- and microRNA-seq

We processed mRNA Illumina Genome Analyzer RNA-seq raw counts covering 507 tumour samples and 60,483 transcripts, and micro-RNA (miRNA) Illumina HighSeq raw counts covering 501 samples and 1,881 miRNAs, using R Programming Language (R) [39]. Transcripts with 0 counts across all samples were removed. Normalisation and variance stabilising transformation were conducted using the DESeq2 package [40], with age at diagnosis, BMI, race, and histologic type included as factors likely to bias counts. Outlier samples were identified by visual observation of principal component 1 (PC1) and PC2. Correlation of clinical parameters to PCs was conducted using a customised level plot function and incorporated Spearman Rank correlation coefficients, with p-values derived by t-test. Differential expression (DE) analysis was conducted using DESeq2 with p-values adjusted by false discovery rate (FDR). Three comparisons between each race and all other races was performed. Quality control (QC) of DE results was conducted using customised functions to generate MA and volcano plots, and also inspection of Cook's distance and plots of mean of normalised counts versus p-value. Clustering with heatmap was performed using the ComplexHeatmap package [41] and included all variance stabilised counts for differentially expressed transcripts at 5% FDR and log2FC>2. Prior to heatmap generation, partitioning around medoids (PAM) was performed on transcripts using default parameters, with defined clusters used to break-up the transcript dendrogram and heatmap. Counts were converted to Z-scores prior to clustering. For transcript and sample dendrograms, Euclidean distance and Ward's linkage were used.

### Somatic mutation and copy number aberrations

Data for a total of 488 patients was available for somatic mutations and 474 patients for somatic CNA (SCNA), respectively. From the somatic mutation data, we filtered out mutations called in a panel of 258 normals used by the Broad Institute (indicated by ‘FILTER’ flag). We included all mutation types in the analysis, including gene coding, UTR, and promoter region and labelled each with HGVS protein ID where available. Recurrent SCNA in the GISTIC 2.0 SCNA data was calculated separately for each race using GAIA [42] by first building a copy number variant (CNV) matrix of regions used by GISTIC 2.0, less known common CNV (available at ftp://ftp.broadinstitute.org/pub/GISTIC2.0/hg19_support/). Recurrent CNV were defined by FDR Q<0.15 using ten iterations. Genomic SCNA plots were generated using a custom R script, with cut-off defined also at FDR Q<0.15. Transcripts overlapping recurrent SCNA were defined using the biomaRt [43] and GenomicRanges [44] packages in R, with “ENSEMBL_MART_ENSEMBL”, “grch37.ensembl.org”, “/biomart/martservice”, and “hsapiens_gene_ensembl” set as the biomart, host, path, and dataset, respectively.

### SCNA groupings

For defining SCNA groups, we performed PAM using the copy number segment mean called per sample in each region that passed FDR Q<0.15. Overlap of each region was performed using GenomicRanges. For Caucasians, we selected a PAM cluster solution of five; for BoAAs, we selected four. Clustering and heatmap generation was performed using the ComplexHeatmap package [41]. Dendrograms were generated using Euclidean distance and Ward's linkage. These groupings were then aligned with the 4 EC categories identified by the TCGA [13] by calculating individual mutation frequencies in each race and each identified SCNA group.

### Overall survival analysis

Kaplan-Meier survival was performed using the survcomp package in R [45]. Overall survival (OS) was compared between each SCNA grouping. Overall survival was defined using the ‘days_to_last_follow-up’ parameter in the TCGA metadata. P values and hazard ratios were derived from a Cox proportional hazards regression model by comparing each curve to the low SCNA group, i.e., the clinically-favourable group, respectively, for BoAA and Caucasians.

## SUPPLEMENTARY MATERIALS FIGURE AND TABLES












