# Supplementary material for: Racial differences in endometrial cancer molecular portraits in The Cancer Genome Atlas
Source: Oncotarget. 2018 Mar 30;9(24):17093–103. doi: 10.18632/oncotarget.24907 (PMC5908308; doi:10.18632/oncotarget.24907)
Supplement: Supplementary file 1 [file oncotarget-09-17093-s001.pdf]

Racial differences in endometrial cancer molecular portraits in The Cancer Genome Atlas

SUPPLEMENTARY MATERIALS

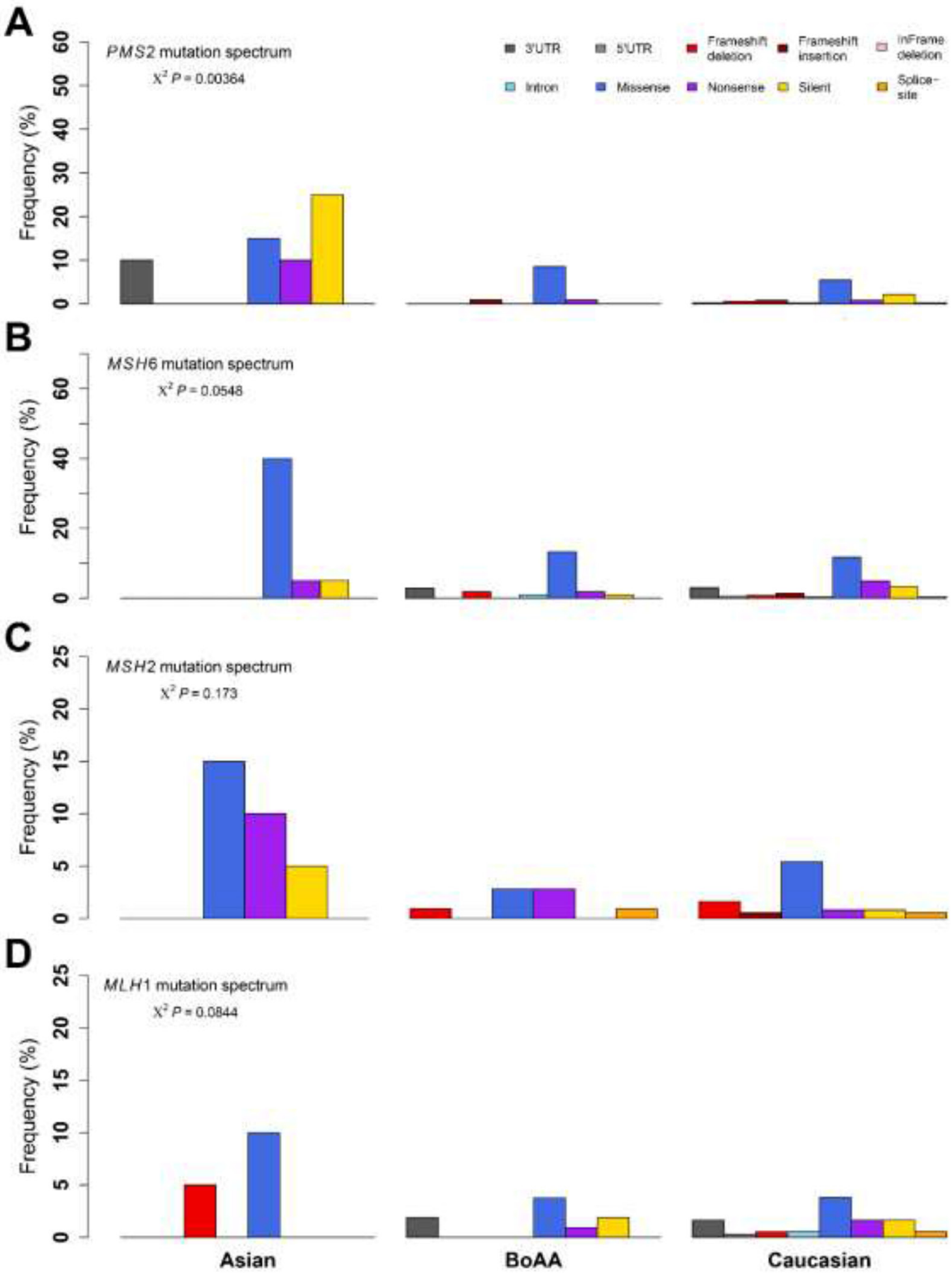

Supplementary Figure 1: (A-D) χ^2 Analysis of genes associated with Lynch syndrome, including *PMS2* (A), *MSH6* (B), *MSH2* (C) and *MLH1* (D). A highly significant enrichment of mutations in each of these genes was shown to be present in Asians.

Supplementary Table 1: Differential expression BoAA Vs Caucasian.

See Supplementary File 1

Supplementary Table 2: Differential expression BoAA Vs Asian.

See Supplementary File 2

Supplementary Table 3: Differential expression Asian Vs Caucasian.

See Supplementary File 3

Supplementary Table 4: Most frequent mutations per race.

See Supplementary File 4

Supplementary Table 5: Individual mutations per SCNA group Caucasian.

See Supplementary File 5
