# Supplementary material for: Racial differences in endometrial cancer molecular portraits in The Cancer Genome Atlas
Source: Oncotarget. 2018 Mar 30;9(24):17093–103. doi: 10.18632/oncotarget.24907 (PMC5908308; doi:10.18632/oncotarget.24907)
Supplement: Supplementary file 2 [file oncotarget-09-17093-s002.doc]

| **Supplementary Table 1: Differential expression BoAA Vs Caucasian** | |  |  |  |  |
| --- | --- | --- | --- | --- | --- |
| **Ensembl** | **HUGO** | **Log2FC** | **Log2FC.SE** | **p.value** | **FDR** |
| ENSG00000171794 | UTF1.protein_coding | 4.47 | 0.37 | 2.76E-34 | 1.78E-30 |
| ENSG00000253239 | IGLVI-70.IG_V_pseudogene | 4.13 | 0.35 | 1.77E-31 | 8.52E-28 |
| ENSG00000145626 | UGT3A1.protein_coding | 3.24 | 0.37 | 7.16E-19 | 1.43E-15 |
| ENSG00000197549 | PRAMENP.transcribed_unprocessed_pseudogene | 2.81 | 0.32 | 1.43E-18 | 2.52E-15 |
| ENSG00000197177 | ADGRA1.protein_coding | 3.37 | 0.39 | 9.03E-18 | 1.38E-14 |
| ENSG00000279338 | ENSG00000279338 | 2.04 | 0.25 | 1.87E-16 | 2.30E-13 |
| ENSG00000265185 | SNORD3B-1.snoRNA | 2.35 | 0.30 | 3.55E-15 | 3.21E-12 |
| ENSG00000188373 | C10orf99.protein_coding | -2.06 | 0.28 | 1.52E-13 | 9.36E-11 |
| ENSG00000168269 | FOXI1.protein_coding | -2.58 | 0.35 | 2.74E-13 | 1.50E-10 |
| ENSG00000160182 | TFF1.protein_coding | -2.27 | 0.31 | 2.94E-13 | 1.59E-10 |
| ENSG00000110484 | SCGB2A2.protein_coding | -2.43 | 0.33 | 3.03E-13 | 1.62E-10 |
| ENSG00000263820 | AC005702.2.miRNA | -2.39 | 0.33 | 4.29E-13 | 2.22E-10 |
| ENSG00000228496 | AC106875.1.sense_intronic | -2.52 | 0.35 | 6.27E-13 | 3.13E-10 |
| ENSG00000256618 | MTRNR2L1.protein_coding | -2.16 | 0.30 | 9.27E-13 | 4.47E-10 |
| ENSG00000243264 | IGKV2D-29.IG_V_gene | 2.34 | 0.33 | 1.93E-12 | 8.47E-10 |
| ENSG00000168515 | SCGB1D1.protein_coding | -2.45 | 0.36 | 5.77E-12 | 2.17E-09 |
| ENSG00000187689 | AMTN.protein_coding | -3.41 | 0.51 | 2.32E-11 | 7.58E-09 |
| ENSG00000230798 | FOXD3-AS1.antisense | 2.29 | 0.34 | 2.78E-11 | 8.91E-09 |
| ENSG00000100604 | CHGA.protein_coding | -2.04 | 0.31 | 3.47E-11 | 1.06E-08 |
| ENSG00000185479 | KRT6B.protein_coding | -2.04 | 0.31 | 4.45E-11 | 1.33E-08 |
| ENSG00000126233 | SLURP1.protein_coding | -2.04 | 0.32 | 1.01E-10 | 2.68E-08 |
| ENSG00000143556 | S100A7.protein_coding | -2.28 | 0.36 | 2.26E-10 | 5.50E-08 |
| ENSG00000170373 | CST1.protein_coding | -2.07 | 0.34 | 1.13E-09 | 2.13E-07 |
| ENSG00000163216 | SPRR2D.protein_coding | -2.37 | 0.42 | 1.86E-08 | 2.27E-06 |
| ENSG00000184608 | FAM167A-AS1.antisense | 2.09 | 0.38 | 2.45E-08 | 2.81E-06 |
| ENSG00000163586 | FABP1.protein_coding | -2.83 | 0.51 | 3.19E-08 | 3.48E-06 |
| ENSG00000260042 | CTD-2034I21.1.lincRNA | -2.49 | 0.47 | 9.46E-08 | 8.67E-06 |
| ENSG00000163209 | SPRR3.protein_coding | -2.14 | 0.42 | 2.49E-07 | 1.89E-05 |
| ENSG00000224899 | RP11-3B12.5.lincRNA | 2.59 | 0.51 | 3.23E-07 | 2.35E-05 |
| ENSG00000229339 | RP11-193I22.2.processed_pseudogene | -2.27 | 0.44 | 3.29E-07 | 2.38E-05 |
| ENSG00000200741 | RNA5SP161.rRNA | -2.87 | 0.57 | 4.60E-07 | 3.04E-05 |
| ENSG00000106331 | PAX4.protein_coding | -2.46 | 0.49 | 5.86E-07 | 3.62E-05 |
| ENSG00000156920 | ADGRG4.protein_coding | -2.15 | 0.44 | 1.04E-06 | 5.67E-05 |
| ENSG00000253301 | LINC01606.lincRNA | 2.02 | 0.42 | 1.12E-06 | 6.04E-05 |
| ENSG00000202187 | RNA5SP355.rRNA | -3.96 | 0.82 | 1.18E-06 | 6.25E-05 |
| ENSG00000071991 | CDH19.protein_coding | 2.25 | 0.47 | 1.79E-06 | 8.83E-05 |
| ENSG00000227094 | RP11-565P22.2.antisense | 2.10 | 0.45 | 2.62E-06 | 1.19E-04 |
| ENSG00000249357 | RP11-432M8.8.processed_pseudogene | -2.02 | 0.45 | 5.80E-06 | 2.20E-04 |
| ENSG00000278099 | U1.snRNA | -2.14 | 0.48 | 7.31E-06 | 2.64E-04 |
| ENSG00000116726 | PRAMEF12.protein_coding | -2.15 | 0.48 | 8.97E-06 | 3.11E-04 |
| ENSG00000136696 | IL36B.protein_coding | -2.43 | 0.55 | 1.08E-05 | 3.56E-04 |
| ENSG00000164816 | DEFA5.protein_coding | -3.35 | 0.80 | 2.88E-05 | 7.77E-04 |
| ENSG00000268799 | RP11-321E2.13.processed_pseudogene | -2.01 | 0.50 | 5.45E-05 | 1.27E-03 |
| ENSG00000272351 | RNA5SP506.rRNA | -2.00 | 0.53 | 1.38E-04 | 2.61E-03 |
