# Supplementary material for: Racial differences in endometrial cancer molecular portraits in The Cancer Genome Atlas
Source: Oncotarget. 2018 Mar 30;9(24):17093–103. doi: 10.18632/oncotarget.24907 (PMC5908308; doi:10.18632/oncotarget.24907)
Supplement: Supplementary file 3 [file oncotarget-09-17093-s003.doc]

| **Supplementary Table 2: Differential expression BoAA Vs Asian** | |  |  |  |  |
| --- | --- | --- | --- | --- | --- |
| **Ensembl** | **HUGO** | **Log2FC** | **Log2FC.SE** | **p.value** | **FDR** |
| ENSG00000132874 | SLC14A2.protein_coding | 4.28 | 0.61 | 1.48E-12 | 1.71E-08 |
| ENSG00000105852 | PON3.protein_coding | -3.33 | 0.49 | 1.48E-11 | 9.49E-08 |
| ENSG00000101842 | VSIG1.protein_coding | -3.01 | 0.47 | 1.37E-10 | 4.42E-07 |
| ENSG00000145626 | UGT3A1.protein_coding | 5.38 | 0.85 | 2.10E-10 | 6.39E-07 |
| ENSG00000243955 | GSTA1.protein_coding | -3.83 | 0.61 | 2.91E-10 | 8.44E-07 |
| ENSG00000157005 | SST.protein_coding | 5.24 | 0.84 | 4.25E-10 | 1.02E-06 |
| ENSG00000230798 | FOXD3-AS1.antisense | 5.09 | 0.81 | 3.97E-10 | 1.02E-06 |
| ENSG00000007350 | TKTL1.protein_coding | 4.28 | 0.68 | 4.20E-10 | 1.02E-06 |
| ENSG00000131471 | AOC3.protein_coding | -2.02 | 0.33 | 1.30E-09 | 2.89E-06 |
| ENSG00000171794 | UTF1.protein_coding | 5.29 | 0.92 | 8.12E-09 | 1.18E-05 |
| ENSG00000090932 | DLL3.protein_coding | 3.21 | 0.56 | 1.05E-08 | 1.44E-05 |
| ENSG00000095713 | CRTAC1.protein_coding | 3.82 | 0.67 | 1.40E-08 | 1.77E-05 |
| ENSG00000279338 | ENSG00000279338 | 3.11 | 0.57 | 5.75E-08 | 6.29E-05 |
| ENSG00000166736 | HTR3A.protein_coding | 3.54 | 0.65 | 5.91E-08 | 6.33E-05 |
| ENSG00000227195 | MIR663AHG.lincRNA | 6.03 | 1.13 | 1.00E-07 | 9.83E-05 |
| ENSG00000104722 | NEFM.protein_coding | -2.71 | 0.51 | 1.16E-07 | 1.07E-04 |
| ENSG00000204511 | MCCD1.protein_coding | 4.69 | 0.91 | 2.54E-07 | 1.99E-04 |
| ENSG00000124260 | MAGEA10.protein_coding | 6.44 | 1.25 | 2.77E-07 | 2.08E-04 |
| ENSG00000119915 | ELOVL3.protein_coding | 2.55 | 0.50 | 3.15E-07 | 2.28E-04 |
| ENSG00000198914 | POU3F3.protein_coding | 5.00 | 0.99 | 4.05E-07 | 2.79E-04 |
| ENSG00000263711 | RP11-169F17.1.lincRNA | 5.83 | 1.16 | 4.50E-07 | 2.93E-04 |
| ENSG00000259803 | SLC22A31.protein_coding | 2.91 | 0.58 | 4.69E-07 | 3.02E-04 |
| ENSG00000241158 | ADAMTS9-AS1.antisense | 2.49 | 0.50 | 7.91E-07 | 4.40E-04 |
| ENSG00000198883 | PNMA5.protein_coding | 4.23 | 0.86 | 8.65E-07 | 4.68E-04 |
| ENSG00000185247 | MAGEA11.protein_coding | 4.89 | 1.01 | 1.44E-06 | 6.72E-04 |
| ENSG00000129988 | LBP.protein_coding | 3.83 | 0.80 | 1.46E-06 | 6.72E-04 |
| ENSG00000197616 | MYH6.protein_coding | 3.45 | 0.72 | 1.61E-06 | 7.20E-04 |
| ENSG00000260159 | RP11-483E23.4.unprocessed_pseudogene | 4.03 | 0.85 | 1.94E-06 | 8.32E-04 |
| ENSG00000211638 | IGLV8-61.IG_V_gene | 3.06 | 0.64 | 1.93E-06 | 8.32E-04 |
| ENSG00000240668 | KRT8P36.processed_pseudogene | -2.50 | 0.53 | 2.76E-06 | 1.09E-03 |
| ENSG00000186838 | SELV.protein_coding | 3.35 | 0.72 | 3.21E-06 | 1.24E-03 |
| ENSG00000266968 | RP11-116O18.1.sense_intronic | 3.63 | 0.78 | 3.42E-06 | 1.28E-03 |
| ENSG00000260105 | AOC4P.transcribed_unprocessed_pseudogene | -2.01 | 0.44 | 4.22E-06 | 1.49E-03 |
| ENSG00000262061 | RP11-1260E13.4.antisense | 2.70 | 0.59 | 4.36E-06 | 1.52E-03 |
| ENSG00000152932 | RAB3C.protein_coding | -2.24 | 0.49 | 4.66E-06 | 1.59E-03 |
| ENSG00000117148 | ACTL8.protein_coding | 3.19 | 0.70 | 4.71E-06 | 1.60E-03 |
| ENSG00000110680 | CALCA.protein_coding | 4.04 | 0.88 | 4.91E-06 | 1.64E-03 |
| ENSG00000233254 | RPL21P134.processed_pseudogene | 2.93 | 0.65 | 5.87E-06 | 1.80E-03 |
| ENSG00000273079 | GRIN2B.protein_coding | 2.76 | 0.62 | 7.67E-06 | 2.18E-03 |
| ENSG00000101210 | EEF1A2.protein_coding | 3.17 | 0.71 | 9.04E-06 | 2.45E-03 |
| ENSG00000266964 | FXYD1.protein_coding | 2.15 | 0.48 | 9.15E-06 | 2.46E-03 |
| ENSG00000189127 | ANKRD34B.protein_coding | 2.64 | 0.60 | 9.68E-06 | 2.57E-03 |
| ENSG00000071991 | CDH19.protein_coding | 5.04 | 1.14 | 1.06E-05 | 2.78E-03 |
| ENSG00000183454 | GRIN2A.protein_coding | 2.46 | 0.56 | 1.15E-05 | 2.93E-03 |
| ENSG00000130829 | DUSP9.protein_coding | 2.47 | 0.56 | 1.21E-05 | 2.99E-03 |
| ENSG00000164530 | PI16.protein_coding | -2.41 | 0.56 | 1.36E-05 | 3.29E-03 |
| ENSG00000253239 | IGLVI-70.IG_V_pseudogene | 3.47 | 0.80 | 1.61E-05 | 3.74E-03 |
| ENSG00000113889 | KNG1.protein_coding | 3.31 | 0.77 | 1.70E-05 | 3.87E-03 |
| ENSG00000265933 | LINC00668.lincRNA | 2.90 | 0.68 | 1.72E-05 | 3.87E-03 |
| ENSG00000185737 | NRG3.protein_coding | 2.97 | 0.69 | 1.88E-05 | 4.10E-03 |
| ENSG00000253642 | RP11-317N12.1.lincRNA | 4.82 | 1.13 | 2.17E-05 | 4.58E-03 |
| ENSG00000197177 | ADGRA1.protein_coding | 3.89 | 0.92 | 2.27E-05 | 4.70E-03 |
| ENSG00000203697 | CAPN8.protein_coding | -2.03 | 0.48 | 2.29E-05 | 4.73E-03 |
| ENSG00000253301 | LINC01606.lincRNA | 4.02 | 0.95 | 2.35E-05 | 4.77E-03 |
| ENSG00000132204 | LINC00470.lincRNA | 3.86 | 0.91 | 2.37E-05 | 4.80E-03 |
| ENSG00000215644 | GCGR.protein_coding | 3.20 | 0.76 | 2.67E-05 | 5.06E-03 |
| ENSG00000267594 | CYP4F24P.unprocessed_pseudogene | -2.61 | 0.63 | 2.90E-05 | 5.34E-03 |
| ENSG00000125804 | FAM182A.lincRNA | 2.23 | 0.53 | 3.18E-05 | 5.61E-03 |
| ENSG00000111700 | SLCO1B3.protein_coding | -3.43 | 0.82 | 3.18E-05 | 5.61E-03 |
| ENSG00000233421 | RP5-875O13.1.lincRNA | 3.10 | 0.75 | 3.57E-05 | 6.12E-03 |
| ENSG00000205628 | LINC01446.lincRNA | 5.07 | 1.24 | 4.07E-05 | 6.72E-03 |
| ENSG00000136244 | IL6.protein_coding | 2.03 | 0.50 | 4.18E-05 | 6.80E-03 |
| ENSG00000228549 | RP11-108M9.3.lincRNA | 2.80 | 0.69 | 4.44E-05 | 7.14E-03 |
| ENSG00000226673 | LINC01108.lincRNA | 2.79 | 0.69 | 4.83E-05 | 7.50E-03 |
| ENSG00000231290 | APCDD1L-AS1.lincRNA | 2.32 | 0.58 | 5.85E-05 | 8.58E-03 |
| ENSG00000184608 | FAM167A-AS1.antisense | 3.78 | 0.94 | 6.18E-05 | 8.93E-03 |
| ENSG00000132026 | RTBDN.protein_coding | 2.30 | 0.58 | 6.64E-05 | 9.39E-03 |
| ENSG00000236502 | SIX3-AS1.lincRNA | 2.99 | 0.76 | 7.46E-05 | 1.02E-02 |
| ENSG00000225329 | LHFPL3-AS2.lincRNA | 2.11 | 0.53 | 7.81E-05 | 1.05E-02 |
| ENSG00000234068 | PAGE2.protein_coding | 4.41 | 1.12 | 8.20E-05 | 1.10E-02 |
| ENSG00000129152 | MYOD1.protein_coding | 3.98 | 1.02 | 9.23E-05 | 1.18E-02 |
| ENSG00000168671 | UGT3A2.protein_coding | 2.44 | 0.63 | 9.26E-05 | 1.18E-02 |
| ENSG00000080293 | SCTR.protein_coding | 2.24 | 0.58 | 9.94E-05 | 1.24E-02 |
| ENSG00000253818 | IGLV1-41.IG_V_pseudogene | 3.35 | 0.86 | 1.01E-04 | 1.25E-02 |
| ENSG00000171873 | ADRA1D.protein_coding | 2.16 | 0.56 | 1.06E-04 | 1.30E-02 |
| ENSG00000177468 | OLIG3.protein_coding | 3.39 | 0.88 | 1.10E-04 | 1.31E-02 |
| ENSG00000239265 | CLRN1-AS1.antisense | 3.07 | 0.80 | 1.19E-04 | 1.39E-02 |
| ENSG00000002726 | AOC1.protein_coding | 2.37 | 0.62 | 1.25E-04 | 1.42E-02 |
| ENSG00000156395 | SORCS3.protein_coding | 2.70 | 0.71 | 1.45E-04 | 1.53E-02 |
| ENSG00000110203 | FOLR3.protein_coding | 3.08 | 0.82 | 1.65E-04 | 1.67E-02 |
| ENSG00000236449 | AC018890.6.antisense | 2.74 | 0.73 | 1.66E-04 | 1.67E-02 |
| ENSG00000155622 | XAGE2.protein_coding | 4.70 | 1.25 | 1.69E-04 | 1.68E-02 |
| ENSG00000249853 | HS3ST5.protein_coding | 2.30 | 0.61 | 1.69E-04 | 1.68E-02 |
| ENSG00000101188 | NTSR1.protein_coding | 2.23 | 0.59 | 1.70E-04 | 1.68E-02 |
| ENSG00000075043 | KCNQ2.protein_coding | 3.11 | 0.83 | 1.74E-04 | 1.70E-02 |
| ENSG00000225667 | ENSG00000225667 | 2.84 | 0.76 | 1.75E-04 | 1.71E-02 |
| ENSG00000112319 | EYA4.protein_coding | 2.93 | 0.78 | 1.82E-04 | 1.76E-02 |
| ENSG00000120210 | INSL6.protein_coding | 4.24 | 1.14 | 1.88E-04 | 1.79E-02 |
| ENSG00000163440 | PDCL2.protein_coding | 3.33 | 0.90 | 1.96E-04 | 1.84E-02 |
| ENSG00000165828 | PRAP1.protein_coding | 2.13 | 0.57 | 2.11E-04 | 1.95E-02 |
| ENSG00000164756 | SLC30A8.protein_coding | 2.92 | 0.79 | 2.19E-04 | 1.99E-02 |
| ENSG00000175426 | PCSK1.protein_coding | 2.24 | 0.61 | 2.24E-04 | 2.00E-02 |
| ENSG00000179930 | ZNF648.protein_coding | 2.08 | 0.56 | 2.29E-04 | 2.03E-02 |
| ENSG00000198681 | MAGEA1.protein_coding | 5.24 | 1.43 | 2.41E-04 | 2.10E-02 |
| ENSG00000204347 | BTBD17.protein_coding | 3.60 | 0.98 | 2.48E-04 | 2.14E-02 |
| ENSG00000116194 | ANGPTL1.protein_coding | 2.01 | 0.55 | 2.54E-04 | 2.19E-02 |
| ENSG00000231870 | KRT17P3.unprocessed_pseudogene | 3.02 | 0.83 | 2.64E-04 | 2.23E-02 |
| ENSG00000236253 | SLC25A3P1.transcribed_processed_pseudogene | 3.54 | 0.98 | 2.97E-04 | 2.42E-02 |
| ENSG00000235621 | LINC00494.lincRNA | 2.50 | 0.69 | 2.97E-04 | 2.42E-02 |
| ENSG00000226057 | PHF2P2.transcribed_unprocessed_pseudogene | 3.11 | 0.86 | 3.18E-04 | 2.55E-02 |
| ENSG00000157851 | DPYSL5.protein_coding | 2.91 | 0.81 | 3.42E-04 | 2.68E-02 |
| ENSG00000166391 | MOGAT2.protein_coding | -2.33 | 0.65 | 3.43E-04 | 2.68E-02 |
| ENSG00000100341 | PNPLA5.protein_coding | 2.28 | 0.64 | 3.44E-04 | 2.68E-02 |
| ENSG00000249413 | RP11-25H12.1.lincRNA | 3.66 | 1.02 | 3.56E-04 | 2.73E-02 |
| ENSG00000258354 | MIR3180-1.lincRNA | 2.15 | 0.60 | 3.56E-04 | 2.73E-02 |
| ENSG00000279220 | GPR1-AS.antisense | 3.42 | 0.96 | 3.86E-04 | 2.86E-02 |
| ENSG00000131668 | BARX1.protein_coding | 2.36 | 0.67 | 3.97E-04 | 2.92E-02 |
| ENSG00000147381 | MAGEA4.protein_coding | 4.36 | 1.24 | 4.21E-04 | 3.04E-02 |
| ENSG00000158022 | TRIM63.protein_coding | 2.03 | 0.58 | 4.22E-04 | 3.05E-02 |
| ENSG00000198910 | L1CAM.protein_coding | 2.25 | 0.64 | 4.25E-04 | 3.06E-02 |
| ENSG00000184350 | MRGPRE.protein_coding | 2.63 | 0.75 | 4.32E-04 | 3.09E-02 |
| ENSG00000171102 | OBP2B.protein_coding | 3.16 | 0.90 | 4.35E-04 | 3.11E-02 |
| ENSG00000101746 | NOL4.protein_coding | 2.32 | 0.66 | 4.38E-04 | 3.12E-02 |
| ENSG00000182111 | ZNF716.protein_coding | 3.87 | 1.10 | 4.44E-04 | 3.14E-02 |
| ENSG00000258602 | RP11-7F17.7.lincRNA | 2.31 | 0.66 | 4.45E-04 | 3.14E-02 |
| ENSG00000249138 | SLED1.transcribed_processed_pseudogene | 2.16 | 0.61 | 4.43E-04 | 3.14E-02 |
| ENSG00000261122 | FLJ26245.lincRNA | 3.31 | 0.96 | 5.83E-04 | 3.76E-02 |
| ENSG00000138083 | SIX3.protein_coding | 2.37 | 0.69 | 5.98E-04 | 3.81E-02 |
| ENSG00000267420 | RP11-527L4.6.lincRNA | 3.31 | 0.97 | 6.05E-04 | 3.83E-02 |
| ENSG00000173908 | KRT28.protein_coding | 4.46 | 1.31 | 6.52E-04 | 4.00E-02 |
| ENSG00000149575 | SCN2B.protein_coding | 2.03 | 0.60 | 6.62E-04 | 4.06E-02 |
| ENSG00000085465 | OVGP1.protein_coding | 2.44 | 0.72 | 6.65E-04 | 4.06E-02 |
| ENSG00000070886 | EPHA8.protein_coding | 2.50 | 0.74 | 7.01E-04 | 4.20E-02 |
| ENSG00000255794 | RMST.lincRNA | 2.72 | 0.81 | 7.54E-04 | 4.35E-02 |
| ENSG00000197888 | UGT2B17.protein_coding | 2.60 | 0.77 | 7.56E-04 | 4.35E-02 |
| ENSG00000197172 | MAGEA6.protein_coding | 4.15 | 1.23 | 7.65E-04 | 4.39E-02 |
| ENSG00000184029 | DSCR4.protein_coding | 3.17 | 0.94 | 7.79E-04 | 4.45E-02 |
| ENSG00000188582 | PAQR9.protein_coding | 2.57 | 0.77 | 7.91E-04 | 4.50E-02 |
| ENSG00000276093 | RP5-1017F8.2.lincRNA | 2.96 | 0.88 | 8.17E-04 | 4.59E-02 |
| ENSG00000229967 | RP11-366F6.2.antisense | 4.99 | 1.49 | 8.50E-04 | 4.69E-02 |
| ENSG00000273677 | WT1-AS_2.misc_RNA | 2.74 | 0.83 | 9.33E-04 | 4.97E-02 |
