# Supplementary material for: Racial differences in endometrial cancer molecular portraits in The Cancer Genome Atlas
Source: Oncotarget. 2018 Mar 30;9(24):17093–103. doi: 10.18632/oncotarget.24907 (PMC5908308; doi:10.18632/oncotarget.24907)
Supplement: Supplementary file 4 [file oncotarget-09-17093-s004.doc]

| **Supplementary Table 3: Differential expression Asian Vs Caucasian** | |  |  |  |  |
| --- | --- | --- | --- | --- | --- |
| **Ensembl** | **HUGO** | **Log2FC** | **Log2FC.SE** | **p.value** | **FDR** |
| ENSG00000243955 | GSTA1.protein_coding | 4.13 | 0.57 | 6.01E-13 | 1.74E-08 |
| ENSG00000101842 | VSIG1.protein_coding | 2.93 | 0.44 | 3.42E-11 | 4.96E-07 |
| ENSG00000104722 | NEFM.protein_coding | 3.16 | 0.48 | 5.64E-11 | 5.67E-07 |
| ENSG00000105852 | PON3.protein_coding | 2.75 | 0.46 | 2.67E-09 | 1.55E-05 |
| ENSG00000198914 | POU3F3.protein_coding | -5.49 | 0.93 | 3.94E-09 | 1.73E-05 |
| ENSG00000110680 | CALCA.protein_coding | -4.73 | 0.84 | 1.52E-08 | 5.17E-05 |
| ENSG00000197888 | UGT2B17.protein_coding | -3.95 | 0.73 | 6.25E-08 | 1.45E-04 |
| ENSG00000168269 | FOXI1.protein_coding | -4.25 | 0.79 | 8.58E-08 | 1.84E-04 |
| ENSG00000267594 | CYP4F24P.unprocessed_pseudogene | 3.14 | 0.59 | 9.38E-08 | 1.94E-04 |
| ENSG00000160181 | TFF2.protein_coding | -3.77 | 0.72 | 1.35E-07 | 2.45E-04 |
| ENSG00000171102 | OBP2B.protein_coding | -4.41 | 0.85 | 2.06E-07 | 3.62E-04 |
| ENSG00000172551 | MUCL1.protein_coding | -3.99 | 0.79 | 4.33E-07 | 6.33E-04 |
| ENSG00000256618 | MTRNR2L1.protein_coding | -3.25 | 0.65 | 6.03E-07 | 8.12E-04 |
| ENSG00000163286 | ALPPL2.protein_coding | -3.08 | 0.63 | 1.08E-06 | 1.26E-03 |
| ENSG00000080293 | SCTR.protein_coding | -2.64 | 0.54 | 1.21E-06 | 1.35E-03 |
| ENSG00000166736 | HTR3A.protein_coding | -3.00 | 0.62 | 1.21E-06 | 1.35E-03 |
| ENSG00000225329 | LHFPL3-AS2.lincRNA | -2.44 | 0.50 | 1.32E-06 | 1.43E-03 |
| ENSG00000185247 | MAGEA11.protein_coding | -4.60 | 0.96 | 1.62E-06 | 1.62E-03 |
| ENSG00000273079 | GRIN2B.protein_coding | -2.79 | 0.59 | 1.82E-06 | 1.76E-03 |
| ENSG00000231290 | APCDD1L-AS1.lincRNA | -2.59 | 0.55 | 2.14E-06 | 1.94E-03 |
| ENSG00000137090 | DMRT1.protein_coding | -2.51 | 0.53 | 2.46E-06 | 2.16E-03 |
| ENSG00000198883 | PNMA5.protein_coding | -3.87 | 0.82 | 2.64E-06 | 2.25E-03 |
| ENSG00000129988 | LBP.protein_coding | -3.50 | 0.75 | 3.26E-06 | 2.68E-03 |
| ENSG00000263711 | RP11-169F17.1.lincRNA | -5.22 | 1.12 | 3.29E-06 | 2.68E-03 |
| ENSG00000117148 | ACTL8.protein_coding | -3.05 | 0.66 | 3.54E-06 | 2.81E-03 |
| ENSG00000234068 | PAGE2.protein_coding | -4.95 | 1.08 | 4.28E-06 | 3.14E-03 |
| ENSG00000131668 | BARX1.protein_coding | -2.86 | 0.63 | 5.31E-06 | 3.77E-03 |
| ENSG00000143452 | HORMAD1.protein_coding | -3.09 | 0.68 | 6.17E-06 | 4.22E-03 |
| ENSG00000183454 | GRIN2A.protein_coding | -2.39 | 0.53 | 7.49E-06 | 4.91E-03 |
| ENSG00000174899 | PQLC2L.protein_coding | -3.16 | 0.71 | 8.14E-06 | 5.13E-03 |
| ENSG00000124260 | MAGEA10.protein_coding | -5.44 | 1.22 | 8.29E-06 | 5.16E-03 |
| ENSG00000095713 | CRTAC1.protein_coding | -2.83 | 0.64 | 8.58E-06 | 5.21E-03 |
| ENSG00000227195 | MIR663AHG.lincRNA | -4.96 | 1.12 | 8.68E-06 | 5.21E-03 |
| ENSG00000101441 | CST4.protein_coding | -3.53 | 0.80 | 8.91E-06 | 5.26E-03 |
| ENSG00000146013 | GFRA3.protein_coding | -2.54 | 0.57 | 9.17E-06 | 5.37E-03 |
| ENSG00000101210 | EEF1A2.protein_coding | -2.95 | 0.67 | 1.24E-05 | 6.84E-03 |
| ENSG00000157005 | SST.protein_coding | -3.46 | 0.79 | 1.26E-05 | 6.90E-03 |
| ENSG00000163440 | PDCL2.protein_coding | -3.70 | 0.85 | 1.36E-05 | 7.12E-03 |
| ENSG00000204511 | MCCD1.protein_coding | -3.77 | 0.87 | 1.40E-05 | 7.23E-03 |
| ENSG00000161055 | SCGB3A1.protein_coding | -2.71 | 0.63 | 1.44E-05 | 7.33E-03 |
| ENSG00000075043 | KCNQ2.protein_coding | -3.36 | 0.78 | 1.73E-05 | 8.35E-03 |
| ENSG00000259803 | SLC22A31.protein_coding | -2.33 | 0.55 | 2.11E-05 | 9.79E-03 |
| ENSG00000181433 | SAGE1.protein_coding | -3.86 | 0.91 | 2.30E-05 | 1.04E-02 |
| ENSG00000163064 | EN1.protein_coding | -3.75 | 0.90 | 3.10E-05 | 1.34E-02 |
| ENSG00000101746 | NOL4.protein_coding | -2.59 | 0.63 | 3.51E-05 | 1.50E-02 |
| ENSG00000188162 | OTOG.protein_coding | -2.25 | 0.55 | 3.73E-05 | 1.54E-02 |
| ENSG00000138083 | SIX3.protein_coding | -2.69 | 0.65 | 3.89E-05 | 1.60E-02 |
| ENSG00000132874 | SLC14A2.protein_coding | -2.35 | 0.57 | 4.16E-05 | 1.65E-02 |
| ENSG00000204421 | LY6G6C.protein_coding | -2.76 | 0.67 | 4.12E-05 | 1.65E-02 |
| ENSG00000261409 | RP6-24A23.7.sense_overlapping | 2.25 | 0.55 | 4.57E-05 | 1.75E-02 |
| ENSG00000078898 | BPIFB2.protein_coding | -3.25 | 0.80 | 4.86E-05 | 1.80E-02 |
| ENSG00000197587 | DMBX1.protein_coding | -2.24 | 0.55 | 5.29E-05 | 1.93E-02 |
| ENSG00000177468 | OLIG3.protein_coding | -3.39 | 0.84 | 5.39E-05 | 1.94E-02 |
| ENSG00000189127 | ANKRD34B.protein_coding | -2.28 | 0.56 | 5.50E-05 | 1.94E-02 |
| ENSG00000165588 | OTX2.protein_coding | -3.83 | 0.95 | 5.48E-05 | 1.94E-02 |
| ENSG00000175868 | CALCB.protein_coding | -2.79 | 0.69 | 6.07E-05 | 2.09E-02 |
| ENSG00000253642 | RP11-317N12.1.lincRNA | -4.54 | 1.13 | 6.25E-05 | 2.13E-02 |
| ENSG00000144481 | TRPM8.protein_coding | -2.02 | 0.51 | 6.76E-05 | 2.26E-02 |
| ENSG00000197616 | MYH6.protein_coding | -2.72 | 0.68 | 6.78E-05 | 2.26E-02 |
| ENSG00000229743 | LINC01159.lincRNA | -4.22 | 1.06 | 6.94E-05 | 2.28E-02 |
| ENSG00000243137 | PSG4.protein_coding | -2.62 | 0.66 | 7.33E-05 | 2.39E-02 |
| ENSG00000134398 | ERN2.protein_coding | -2.15 | 0.55 | 8.00E-05 | 2.56E-02 |
| ENSG00000168878 | SFTPB.protein_coding | -2.65 | 0.67 | 8.45E-05 | 2.69E-02 |
| ENSG00000163207 | IVL.protein_coding | -3.14 | 0.80 | 9.17E-05 | 2.86E-02 |
| ENSG00000164434 | FABP7.protein_coding | -3.71 | 0.95 | 9.84E-05 | 3.03E-02 |
| ENSG00000147381 | MAGEA4.protein_coding | -4.52 | 1.17 | 1.11E-04 | 3.32E-02 |
| ENSG00000142549 | IGLON5.protein_coding | -2.17 | 0.56 | 1.22E-04 | 3.46E-02 |
| ENSG00000164089 | ETNPPL.protein_coding | -2.27 | 0.59 | 1.22E-04 | 3.46E-02 |
| ENSG00000143556 | S100A7.protein_coding | -2.95 | 0.77 | 1.20E-04 | 3.46E-02 |
| ENSG00000225667 | ENSG00000225667 | -3.28 | 0.85 | 1.23E-04 | 3.46E-02 |
| ENSG00000178522 | AMBN.protein_coding | -4.35 | 1.13 | 1.23E-04 | 3.46E-02 |
| ENSG00000085465 | OVGP1.protein_coding | -2.59 | 0.68 | 1.24E-04 | 3.48E-02 |
| ENSG00000261122 | FLJ26245.lincRNA | -3.46 | 0.91 | 1.40E-04 | 3.73E-02 |
| ENSG00000081248 | CACNA1S.protein_coding | -3.03 | 0.80 | 1.66E-04 | 4.21E-02 |
| ENSG00000215644 | GCGR.protein_coding | -2.71 | 0.72 | 1.68E-04 | 4.21E-02 |
| ENSG00000197172 | MAGEA6.protein_coding | -4.37 | 1.17 | 1.83E-04 | 4.52E-02 |
| ENSG00000007350 | TKTL1.protein_coding | -2.42 | 0.65 | 1.89E-04 | 4.63E-02 |
| ENSG00000177984 | LCN15.protein_coding | -2.59 | 0.69 | 1.93E-04 | 4.66E-02 |
