# Supplementary material for: Racial differences in endometrial cancer molecular portraits in The Cancer Genome Atlas
Source: Oncotarget. 2018 Mar 30;9(24):17093–103. doi: 10.18632/oncotarget.24907 (PMC5908308; doi:10.18632/oncotarget.24907)
Supplement: Supplementary file 5 [file oncotarget-09-17093-s005.doc]

| **Supplementary Table 4: Most frequent mutations per race** | | | |  |  |  |  |  |  |  |  |
| --- | --- | --- | --- | --- | --- | --- | --- | --- | --- | --- | --- |
| **Caucasian** | | | | **Asian** | | | | **BoAA** | | | |
| **Gene** | **Type** | **HGVS** | **n (%)** | **Gene** | **Type** | **HGVS** | **n (%)** | **Gene** | **Type** | **HGVS** | **n (%)** |
| *PTEN* | Missense | p.Arg130Gly | 36 (9.94) | *PTEN* | Missense | p.Arg130Gln | 6 (30) | *KRAS* | Missense | p.Gly12Asp | 8 (7.55) |
| *SETD1B* | Frame-shift deletion | p.His8ThrfsTer27 | 35 (9.67) | *PTEN* | Nonsense | p.Arg233Ter | 4 (20) | *RPL22* | Frame-shift deletion | p.Lys15ArgfsTer5 | 7 (6.60) |
| *RPL22* | Frame-shift deletion | p.Lys15ArgfsTer5 | 34 (9.39) | *JAK1* | Frame-shift deletion | p.Lys860AsnfsTer16 | 4 (20) | *JAK1* | Frame-shift deletion | p.Lys860AsnfsTer16 | 7 (6.60) |
| *RNF43* | Frame-shift deletion | p.Gly659ValfsTer41 | 30 (8.29) | *ARID1A* | Nonsense | p.Arg1989Ter | 3 (15) | *SETD1B* | Frame-shift deletion | p.His8ThrfsTer27 | 6 (5.66) |
| *JAK1* | Frame-shift deletion | p.Lys860AsnfsTer16 | 27 (7.46) | *RPL22* | Frame-shift deletion | p.Lys15ArgfsTer5 | 3 (15) | *RNF43* | Frame-shift deletion | p.Gly659ValfsTer41 | 6 (5.66) |
| *PIK3CA* | Missense | p.Arg88Gln | 24 (6.63) | *PQLC2L* | Missense | p.Asp78Asn | 3 (15) | *TP53* | Missense | p.Arg273His | 6 (5.66) |
| *PIK3CA* | Missense | p.His1047Arg | 23 (6.35) | *LTV1* | Nonsense | p.Arg377Ter | 3 (15) | *ACVR2A* | Frame-shift deletion | p.Lys437ArgfsTer5 | 6 (5.66) |
| *DOCK3* | Frame-shift deletion | p.Pro1852GlnfsTer45 | 22 (6.08) | *ABCB5* | Frame-shift insertion | p.Ile217ThrfsTer3 | 3 (15) | *PIK3CA* | Missense | p.Arg88Gln | 6 (5.66) |
| *FGFR2* | Missense | p.Ser252Trp | 21 (5.80) | *SETX* | Missense | p.Arg1606Gln | 3 (15) | *BCOR* | Missense | p.Asn1459Ser | 6 (5.66) |
| *KRAS* | Missense | p.Gly12Asp | 20 (5.52) | *SFXN3* | Missense | p.Arg38Gln | 2 (10) | *SPOP* | Missense | p.Met117Val | 5 (4.72) |
| *PTEN* | Missense | p.Arg130Gln | 19 (5.25) | *CFAP43* | Missense | p.Arg1047Gln | 2 (10) | *SMAD7* | Frame-shift deletion | p.Pro209LeufsTer115 | 5 (4.72) |
| *PTEN* | Nonsense | p.Arg233Ter | 18 (4.97) | *CFAP43* | Missense | p.Ser473Leu | 2 (10) | *SCAF4* | Frame-shift deletion | p.Pro250HisfsTer96 | 5 (4.72) |
| *PPP2R1A* | Missense | p.Pro179Arg | 18 (4.97) | *SHOC2* | Missense | p.Arg200His | 2 (10) | *ESRP1* | Frame-shift deletion | p.Asn512ThrfsTer2 | 5 (4.72) |
| *BCOR* | Missense | p.Asn1459Ser | 18 (4.97) | *GPAM* | Missense | p.Val112Ile | 2 (10) | *PAX2* | Frame-shift deletion | p.Arg403GlyfsTer37 | 4 (3.77) |
| *KRAS* | Missense | p.Gly12Val | 17 (4.70) | *FAM188A* | Missense | p.Ser69Leu | 2 (10) | *PTEN* | Missense | p.Arg130Gln | 4 (3.77) |
| *OR14K1* | Missense | p.Arg14Ser | 17 (4.70) | *HACD1* | Missense | p.Leu252Ile | 2 (10) | *PTEN* | Missense | p.Arg142Trp | 4 (3.77) |
| *CTCF* | Frame-shift insertion | p.Thr204AsnfsTer26 | 16 (4.42) | *ARHGAP21* | Missense | p.Gly1547Val | 2 (10) | *PTEN* | Nonsense | p.Arg233Ter | 4 (3.77) |
| *ZBTB20* | Frame-shift deletion | p.Pro692LeufsTer43 | 15 (4.14) | *ARHGAP21* | Frame-shift insertion | p.Gly1547Ter | 2 (10) | *TRIM51* | Frame-shift deletion | p.Met144CysfsTer15 | 4 (3.77) |
| *SLC3A2* | Frame-shift deletion | p.Lys300ArgfsTer31 | 14 (3.87) | *CCDC7* | Nonsense | p.Glu60Ter | 2 (10) | *INPPL1* | Frame-shift deletion | p.Arg1156GlyfsTer46 | 4 (3.77) |
| *ARID1A* | Nonsense | p.Arg1989Ter | 14 (3.87) | *CREM* | Nonsense | p.Glu80Ter | 2 (10) | *PLEKHA6* | Frame-shift deletion | p.Val328TyrfsTer172 | 4 (3.77) |
| *UPF3A* | Frame-shift deletion | p.Glu267ArgfsTer13 | 14 (3.87) | *ZNF485* | Missense | p.Arg206Ile | 2 (10) | *POLE* | Missense | p.Pro286Arg | 4 (3.77) |
| *ACVR2A* | Frame-shift deletion | p.Lys437ArgfsTer5 | 14 (3.87) | *CTGLF11P* | Missense | p.His132Arg | 2 (10) | *TP53* | Missense | p.Arg273Cys | 4 (3.77) |
| *CTGLF11P* | Missense | p.His132Arg | 13 (3.59) | *ANK3* | Nonsense | p.Arg1176Ter | 2 (10) | *PPP2R1A* | Missense | p.Pro179Arg | 4 (3.77) |
| *CAMSAP2* | Frame-shift deletion | p.Met1437Ter | 13 (3.59) | *RTKN2* | Nonsense | p.Arg33Ter | 2 (10) | *PIK3CA* | Missense | p.Gly118Asp | 4 (3.77) |
| *ELMSAN1* | Frame-shift deletion | p.Asn314ThrfsTer4 | 13 (3.59) | *USP54* | Missense | p.Arg657Gln | 2 (10) | *PIK3CA* | Missense | p.His1047Arg | 4 (3.77) |
