# Supplementary material for: Racial differences in endometrial cancer molecular portraits in The Cancer Genome Atlas
Source: Oncotarget. 2018 Mar 30;9(24):17093–103. doi: 10.18632/oncotarget.24907 (PMC5908308; doi:10.18632/oncotarget.24907)
Supplement: Supplementary file 6 [file oncotarget-09-17093-s006.doc]

| **Supplementary Table 5. Individual mutations per SCNA group** | | | | |  |  |  |  |  |  |  |
| --- | --- | --- | --- | --- | --- | --- | --- | --- | --- | --- | --- |
| **Caucasian** |  |  |  |  |  |  |  |  |  |  |  |
| **GROUP 1 (n=173)** | | | | **GROUP 2 (n=40)** | | | | **GROUP 3 (n=67)** | | | |
| **Gene** | **Type** | **HGVS** | **n (%)** | **Gene** | **Type** | **HGVS** | **n (%)** | **Gene** | **Type** | **HGVS** | **n (%)** |
| *SETD1B* | Frame-shift deletion | p.His8ThrfsTer27 | 28 (16.18) | *FGFR2* | Missense | p.Ser252Trp | 7 (17.5) | *PIK3CA* | Missense | p.His1047Arg | 7 (10.45) |
| *RNF43* | Frame-shift deletion | p.Gly659ValfsTer41 | 27 (15.61) | *PTEN* | Missense | p.Arg130Gly | 6 (15.0) | *PTEN* | Missense | p.Arg130Gly | 6 (8.96) |
| *RPL22* | Frame-shift deletion | p.Lys15ArgfsTer5 | 25 (14.45) | *PIK3CA* | Missense | p.His1047Arg | 4 (10.0) | *CAMSAP2* | Frame-shift deletion | p.Met1437Ter | 6 (8.96) |
| *JAK1* | Frame-shift deletion | p.Lys860AsnfsTer16 | 25 (14.45) | *CTNNB1* | Missense | p.Ser37Cys | 4 (10.0) | *RPL22* | Frame-shift deletion | p.Lys15ArgfsTer5 | 6 (8.96) |
| *PTEN* | Missense | p.Arg130Gly | 23 (13.29) | *PTEN* | Nonsense | p.Arg233Ter | 3 (7.5) | *ACVR2A* | Frame-shift deletion | p.Lys437ArgfsTer5 | 5 (7.46) |
| *PTEN* | Missense | p.Arg130Gln | 17 (9.83) | *KRAS* | Missense | p.Gly12Val | 3 (7.5) | *NIPBL* | Frame-shift deletion | p.Ile2776LeufsTer3 | 5 (7.46) |
| *PIK3CA* | Missense | p.Arg88Gln | 17 (9.83) | *KRAS* | Missense | p.Gly12Cys | 3 (7.5) | *BCOR* | Missense | p.Asn1459Ser | 5 (7.46) |
| *DOCK3* | Frame-shift deletion | p.Pro1852GlnfsTer45 | 15 (8.67) | *OR14K1* | Missense | p.Arg14Ser | 3 (7.5) | *SETD1B* | Frame-shift deletion | p.His8ThrfsTer27 | 4 (5.97) |
| *PTEN* | Nonsense | p.Arg233Ter | 13 (7.51) | *SOS1* | Missense | p.Asn233Tyr | 3 (7.5) | *KRAS* | Missense | p.Gly12Asp | 4 (5.97) |
| *BCOR* | Missense | p.Asn1459Ser | 13 (7.51) | *PIK3R1* | Missense | p.Gly376Arg | 3 (7.5) | *ARID1A* | Nonsense | p.Arg1989Ter | 4 (5.97) |
| *KRAS* | Missense | p.Gly12Asp | 12 (6.94) | *SMC3* | Frame-shift deletion | p.Tyr43MetfsTer69 | 2 (5.0) | *UPF3A* | Frame-shift deletion | p.Glu267ArgfsTer13 | 4 (5.97) |
| *CTCF* | Frame-shift insertion | p.Thr204AsnfsTer26 | 12 (6.94) | *FGFR2* | Missense | p.Cys382Arg | 2 (5.0) | *ZNF609* | Frame-shift deletion | p.Lys734ArgfsTer12 | 4 (5.97) |
| *FGFR2* | Missense | p.Ser252Trp | 11 (6.36) | *MYO3A* | Frame-shift deletion | p.Met1192Ter | 2 (5.0) | *XYLT2* | Frame-shift deletion | p.Gly529AlafsTer78 | 4 (5.97) |
| *ZBTB20* | Frame-shift deletion | p.Pro692LeufsTer43 | 11 (6.36) | *CTGLF11P* | Missense | p.His132Arg | 2 (5.0) | *SMAD7* | Frame-shift deletion | p.Pro209LeufsTer115 | 4 (5.97) |
| *SLC3A2* | Frame_Shift_Del | p.Lys300ArgfsTer31 | 10 (5.78) | *PTEN* | Nonsense | p.Arg130Ter | 2 (5.0) | *BZW1* | Frame-shift deletion | p.Asn285ThrfsTer11 | 4 (5.97) |
| **Caucasian (continued)** |  |  |  |  |  |  |  |  |  |  |  |
| **GROUP 4 (n=39)** | | | | **GROUP 5 (n=49)** | | | |  |  |  |  |
| **Gene** | **Type** | **HGVS** | **n (%)** | **Gene** | **Type** | **HGVS** | **n (%)** |  |  |  |  |
| *TP53* | Missense | p.Cys141Tyr | 3 (7.69) | *PPP2R1A* | Missense | p.Pro179Arg | 12 (24.49) |  |  |  |  |
| *PPP2R1A* | Missense | p.Pro179Arg | 3 (7.69) | *GOLGA6L6* | Missense | p.Glu519Val | 4 (8.16) |  |  |  |  |
| *PIK3CA* | Missense | p.Glu542Lys | 3 (7.69) | *OR14K1* | Missense | p.Arg14Ser | 3 (6.12) |  |  |  |  |
| *FGFR2* | Missense | p.Ser252Trp | 2 (5.13) | *TP53* | Missense | p.Arg273His | 3 (6.12) |  |  |  |  |
| *PTEN* | Missense | p.Arg130Pro | 2 (5.13) | *TP53* | Missense | p.Arg273Cys | 3 (6.12) |  |  |  |  |
| *CHD4* | Missense | p.Arg1162Trp | 2 (5.13) | *TP53* | Missense | p.Tyr220Cys | 3 (6.12) |  |  |  |  |
| *TP53* | Missense | p.Arg282Trp | 2 (5.13) | *PIK3CA* | Missense | p.Glu542Lys | 3 (6.12) |  |  |  |  |
| *TP53* | Missense | p.Arg248Trp | 2 (5.13) | *PCNXL3* | Missense | p.Pro403Ser | 2 (4.08) |  |  |  |  |
| *PPP2R1A* | Missense | p.Ser256Phe | 2 (5.13) | *KRAS* | Missense | p.Gly12Val | 2 (4.08) |  |  |  |  |
| *PPP2R1A* | Missense | p.Trp257Leu | 2 (5.13) | *CHD4* | Missense | p.Arg975His | 2 (4.08) |  |  |  |  |
| *PIK3CA* | Missense | p.Gln546Pro | 2 (5.13) | *GOLGA6L6* | Missense | p.Met472Ile | 2 (4.08) |  |  |  |  |
| *PIK3CA* | Missense | p.His1047Arg | 2 (5.13) | *GOLGA6L6* | Missense | p.Lys425Glu | 2 (4.08) |  |  |  |  |
| *TLX1NB* | Missense | p.His81Arg | 1 (2.56) | *GOLGA6L6* | Missense | p.Glu376Lys | 2 (4.08) |  |  |  |  |
| *POLL* | Missense | p.Pro231Leu | 1 (2.56) | *GOLGA6L9* | Missense | p.Ala373Pro | 2 (4.08) |  |  |  |  |
| *KCNIP2* | Missense | p.Pro44Ser | 1 (2.56) | *TP53* | Missense | p.Arg248Gln | 2 (4.08) |  |  |  |  |
|  |  |  |  |  |  |  |  |  |  |  |  |
| **BoAA** |  |  |  |  |  |  |  |  |  |  |  |
| **GROUP 1 (n=23)** | | | | **GROUP 2 (n=31)** | | | | **GROUP 3 (n=30)** | | | |
| **Gene** | **Type** | **HGVS** | **n (%)** | **Gene** | **Type** | **HGVS** | **n (%)** | **Gene** | **Type** | **HGVS** | **n (%)** |
| *TP53* | Missense | p.Arg273His | 3 (13.04) | *TP53* | Missense | p.Arg273His | 3 (9.68) | *PTEN* | Nonsense | p.Arg233Ter | 4 (13.3) |
| *SPOP* | Missense | p.Met117Val | 2 (8.70) | *KMT2D* | Frame-shift deletion | p.Gly1235ValfsTer95 | 2 (6.45) | *POLE* | Missense | p.Pro286Arg | 4 (13.3) |
| *TP53* | Missense | p.Arg248Gln | 2 (8.70) | *GOLGA6L6* | Missense | p.Met472Ile | 2 (6.45) | *ACVR2A* | Frame-shift deletion | p.Lys437ArgfsTer5 | 4 (13.3) |
| *PPP2R1A* | Missense | p.Pro179Arg | 2 (8.70) | *CHGB* | Missense | p.Val351Ile | 2 (6.45) | *PIK3CA* | Missense | p.Arg88Gln | 4 (13.3) |
| *DSCAM* | Missense | p.Asn1363Lys | 2 (8.70) | *FBXW7* | Missense | p.Arg465His | 2 (6.45) | *BCOR* | Missense | p.Asn1459Ser | 4 (13.3) |
| *FBXW7* | Missense | p.Arg505Gly | 2 (8.70) | *SRP72* | Frame-shift deletion | p.Val9CysfsTer10 | 2 (6.45) | *PTEN* | Missense | p.Arg130Gln | 3 (10.0) |
| *GTPBP4* | Missense | p.Asn234Tyr | 1 (4.35) | *PKD2L1* | Missense | p.Leu374Phe | 1 (3.23) | *PIWIL1* | Missense | p.Arg275Gln | 3 (10.0) |
| *CHUK* | Missense | p.Leu152Phe | 1 (4.35) | *SCD* | Missense | p.Gly31Val | 1 (3.23) | *KRAS* | Missense | p.Gly12Asp | 3 (10.0) |
| *WNT8B* | Missense | p.Arg79His | 1 (4.35) | *NDUFB8* | Missense | p.Asp110His | 1 (3.23) | *DIO1* | Missense | p.Arg241Cys | 3 (10.0) |
| *HPS6* | Missense | p.Asp714His | 1 (4.35) | *FGF8* | Missense | p.Ser138Ile | 1 (3.23) | *RPL22* | Frame-shift deletion | p.Lys15ArgfsTer5 | 3 (10.0) |
| *GBF1* | Missense | p.Gln1467Lys | 1 (4.35) | *IDI2* | Missense | p.Arg142Lys | 1 (3.23) | *CDH1* | Frame-shift deletion | p.Pro126ArgfsTer89 | 3 (10.0) |
| *ACTR1A* | Missense | p.Arg46Cys | 1 (4.35) | *PPRC1* | Missense | p.Gln863His | 1 (3.23) | *NF1* | Nonsense | p.Arg2450Ter | 3 (10.0) |
| *NEURL1* | Missense | p.Ser493Cys | 1 (4.35) | *GBF1* | Missense | p.Ser289Cys | 1 (3.23) | *RNF43* | Frame-shift deletion | p.Gly659ValfsTer41 | 3 (10.0) |
| *SORCS3* | Missense | p.Glu378Asp | 1 (4.35) | *GBF1* | Missense | p.Glu626Asp | 1 (3.23) | *SMAD7* | Frame-shift deletion | p.Pro209LeufsTer115 | 3 (10.0) |
| *SORCS3* | Missense | p.Gln980His | 1 (4.35) | *NFKB2* | Missense | p.Asp561Tyr | 1 (3.23) | *ZNF490* | Missense | p.Arg382Ile | 3 (10.0) |
| **BoAA (cont.)** |  |  |  |  |  |  |  |  |  |  |  |
| **GROUP 4 (n=22)** | | | |  |  |  |  |  |  |  |  |
| **Gene** | **Type** | **HGVS** | **n (%)** |  |  |  |  |  |  |  |  |
| *SETD1B* | Frame-shift deletion | p.His8ThrfsTer27 | 4 (18.18) |  |  |  |  |  |  |  |  |
| *KRAS* | Missense | p.Gly12Asp | 4 (18.18) |  |  |  |  |  |  |  |  |
| *RPL22* | Frame-shift deletion | p.Lys15ArgfsTer5 | 4 (18.18) |  |  |  |  |  |  |  |  |
| *JAK1* | Frame-shift deletion | p.Lys860AsnfsTer16 | 4 (18.18) |  |  |  |  |  |  |  |  |
| *PAX2* | Frame-shift deletion | p.Arg403GlyfsTer37 | 3 (13.64) |  |  |  |  |  |  |  |  |
| *MVK* | Frame-shift deletion | p.Ala141ArgfsTer18 | 3 (13.64) |  |  |  |  |  |  |  |  |
| *ZNF124* | Frame-shift deletion | p.Thr339LeufsTer31 | 3 (13.64) |  |  |  |  |  |  |  |  |
| *KMT2D* | Frame-shift deletion | p.Pro2354LeufsTer30 | 3 (13.64) |  |  |  |  |  |  |  |  |
| *SPECC1* | Frame-shift deletion | p.Asn303ThrfsTer63 | 3 (13.64) |  |  |  |  |  |  |  |  |
| *RNF43* | Frame-shift deletion | p.Gly659ValfsTer41 | 3 (13.64) |  |  |  |  |  |  |  |  |
| *KMT2B* | Frame-shift deletion | p.Lys553AsnfsTer52 | 3 (13.64) |  |  |  |  |  |  |  |  |
| *PHF2* | Frame-shift deletion | p.Lys492ArgfsTer6 | 3 (13.64) |  |  |  |  |  |  |  |  |
| *USP26* | Frame-shift deletion | p.Phe348LeufsTer7 | 3 (13.64) |  |  |  |  |  |  |  |  |
| *TUBGCP2* | Frame-shift deletion | p.Gln883LysfsTer25 | 2 (9.09) |  |  |  |  |  |  |  |  |
| *CTGLF11P* | Missense | p.His132Arg | 2 (9.09) |  |  |  |  |  |  |  |  |
